# Molecular survey of tick-borne infectious agents in cricetid rodents (Rodentia: Cricetidae) in Central and Southern Chile

**DOI:** 10.3389/fvets.2024.1399783

**Published:** 2024-05-17

**Authors:** Marlon Mauricio Ardila, Richard Thomas, Adriana Santodomingo, María C. Silva-de la Fuente, Sebastián Muñoz-Leal, Carlos Landaeta-Aqueveque, AnaLía Henríquez

**Affiliations:** ^1^Facultad de Ciencias Veterinarias, Universidad de Concepción, Chillán, Chile; ^2^Facultad de Ciencias Básicas, Universidad del Atlántico, Puerto Colombia, Colombia; ^3^Facultad de Ciencias Agrarias y Forestales, Universidad Católica del Maule, Curicó, Chile; ^4^Facultad de Ciencias de la Naturaleza, Universidad San Sebastián, Concepción, Chile

**Keywords:** tick-borne disease, wild rodents, genetic screenings, surveillance, South American rodents

## Abstract

Tick-borne infectious agents (TBIAs) include several bacteria and protozoa that can infect vertebrates, including humans. Some of these agents can cause important diseases from both a public health perspective, such as Lyme disease, and from an animal health and production viewpoint, such as Texas fever. In Chile, several studies have assessed the presence of tick-borne disease agents in vectors and mammal hosts, mainly in the northern regions, but few studies have assessed the presence of these agents in Central and Southern Chile. This study aimed to assess the presence of three groups of TBIAs—*Borrelia*, *Anaplasmataceae*, and Piroplasmida—in cricetid rodents of Central and Southern Chile. A total of 207 specimens from 13 localities between the O’Higgins and Los Lagos regions were captured. DNA was extracted from the liver and spleen, and subsequently underwent polymerase chain reaction (PCR) amplification targeting the 16S rRNA, *flaB*, and 18S rRNA genes to detect DNA from *Borrelia*, *Anaplasmataceae*, and Piroplasmida, respectively. Although no DNA from these TBIAs was detected, the DNA extraction process was validated by optimal DNA purity ratios (an A260/A280 ratio within the 1.6–2.0 range) and successful internal control amplification in all samples. These results, in addition to findings from previous reports, suggest a very low prevalence of these TBIAs in the rodent population studied. Further research into the factors influencing the presence of these agents and their vectors will provide insight into the reasons underlying this low prevalence.

## 1 Introduction

Mammals in Chile are represented by nine orders and 150 species, with Rodentia being the order with the highest diversity, comprising 31 genera and 67 native species. This constitutes the highest specific diversity of mammals in Chile, accounting for 61.3% of species richness (1–4). The Cricetidae is the most diverse family of rodents in Chile with 39 species (2). Despite their vast diversity, recent studies of pathogens among rodents in Chile are predominantly focused on searching for gastrointestinal and ectoparasites (5–12), in addition to *Trypanosoma cruzi* (13), and “*Candidatus* Orientia chiloensis” infection (14, 15), with few studies on *Borrelia* (16, 17), *Anaplasmataceae* (18), and Piroplasmida (19).

*Borrelia* is a genus of spirochete bacteria, including both pathogenic and non-pathogenic species that affect a variety of hosts, primarily transmitted by ticks. *Borrelia burgdorferi sensu stricto, Borrelia afzelii*, and *Borrelia garinii* are species that are known to cause Lyme disease in humans in the northern hemisphere; this disease is primarily transmitted by ticks of the genus *Ixodes* (20). Other *Borrelia* species are primarily associated with *Ornithodoros* spp. and are the agents of tick-borne relapsing fever in humans (21).

*Anaplasmataceae* is a family of obligate intracellular gram-negative alphaproteobacteria belonging to *Rickettsiales*. These bacteria can infect ticks and vertebrates, and multiply within host cells, typically in leukocytes or endothelial cells of the blood vessels (22, 23). Diseases caused by bacteria of the *Anaplasmataceae* family affect humans and animals, and their geographic distribution is often linked to arthropod vectors (22, 23). This family includes *Anaplasma, Ehrlichia, Neorickettsia,* “*Candidatus* Neoehrlichia,” and *Wolbachia*, among other genera (24).

The order Piroplasmida comprises a group of obligate intracellular parasitic protozoa belonging to the phylum Apicomplexa. Ticks transmit these organisms and affect mammals and birds (25, 26). A prominent genus within Piroplasmida is *Babesia*, which includes various species with distinctive characteristics. *Babesia microti* is a major cause of human babesiosis in regions such as the Nearctic, while *Babesia divergens* and *Babesia bovis* affect bovine livestock in Europe and the Neotropics, respectively (25, 26). Genetic variability and host-specific adaptations present challenges in understanding those diseases.

Rodents play a crucial role as reservoir hosts. At least 2,017 of the 2,777 rodent species described harbor 66 zoonotic agents (27, 28), and cricetid rodents exhibit a higher propensity to carry infectious agents with zoonotic potential (28). Thus, considering the need to understand the distribution of these tick-borne infectious agents (TBIAs) in cricetid rodents in Chile, the objective of this study was to assess the presence of *Borrelia*, *Anaplasmataceae*, and Piroplasmida in cricetid rodents in Central and Southern Chile.

## 2 Materials and methods

### 2.1 Study area and rodent collection

**Figure 1:**
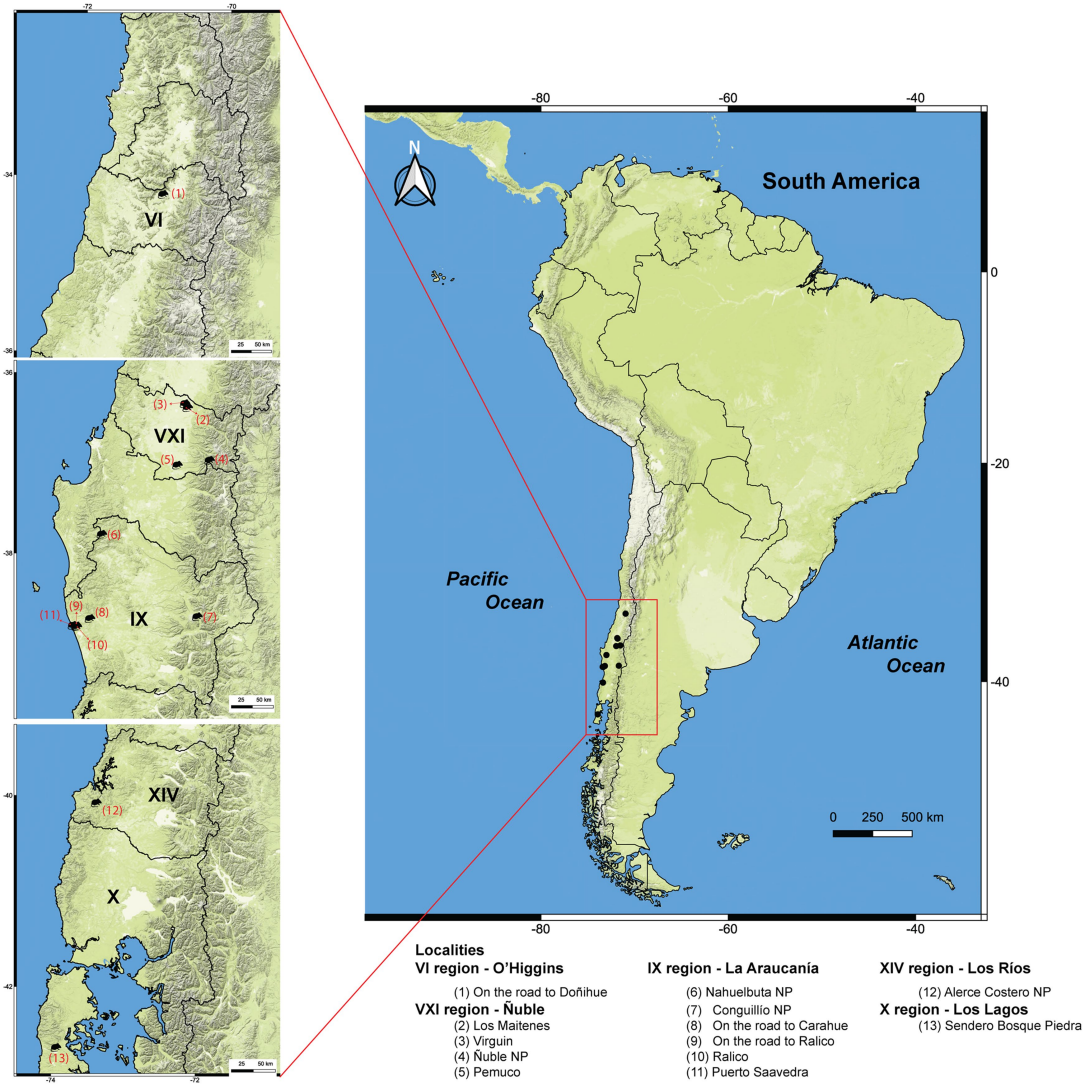
Map of Chile illustrating the localities of rodent collections, indicated by red numerals. Administrative regions are labeled with Roman numerals. NP = National Park. The maps were constructed using the Quantum Geographic Information System (QGIS) 3.18.1-Zürich (https://www.gnu.org/licenses). Map Layer was obtained from OpenStreetMap®, which is licensed under the Open Data Commons Open Database License (ODbL) by the OpenStreetMap Foundation (OSMF). Rodent silhouettes were obtained from the PhyloPic portal (https://www.phylopic.org/) (accessed on 28 Jan 2024).

Collections were performed from 2017 to 2019 in 13 localities in Central and Southern Chile between latitudes 34 and 43°S (Figure 1). This area encompasses climates ranging from Mediterranean in the Ñuble National Reserve to temperate oceanic in the other localities, with increasing humidity and decreasing temperatures as one moves southward. Trapping and euthanasia were performed as previously described (29) and according to the American Veterinary Medical Association Guidelines for the Euthanasia of Animals 2020 (30). Liver and spleen samples were preserved in 95% ethanol for up to 20 days and kept at −20°C until DNA extraction process, 1–3 months later. The number of captured specimens per species was based on capture permits granted by Chilean authorities; likewise, this number was also constrained by trapping success, and all captures adhered to Chilean legislation (31).

### 2.2 DNA extraction and gene amplification

Genomic DNA was extracted from organ samples using the DNeasy Blood & Tissue Kit™ (QIAGEN^®^, Hilden, Germany) according to the manufacturer’s protocol and eluted in 40 μL of buffer AE (10 mM Tris–Cl; 0.5 mM ethylenediaminetetraacetic acid [EDTA], pH 9.0). DNA concentration was quantified with an Epoch™ Microplate Spectrophotometer (BioTek Instruments, Inc., Winooski, VT, United States), and quality of the sample was checked by means of the A260/280 ratio according to Khare et al. (32). Successful DNA extractions were confirmed through conventional polymerase chain reaction (PCR) targeting the mammalian glyceraldehyde-3-phosphate dehydrogenase (*GAPDH*) gene (33). *GAPDH*-positive samples were subsequently used for genetic screenings for *Borrelia*, *Anaplasmataceae*, and Piroplasmida using molecular markers, primers, and thermal conditions stated in Supplementary Table S1. PCRs were performed in a thermal cycler ProFlex™ Base 32 × 3 (Applied Biosystems, Thermo Fisher Scientific, Waltham, MA, United States) using 25 μL of the reaction mixtures containing the following proportions: 12.5 μL DreamTaq Green PCR Master Mix (Thermo Fisher Scientific), 1 μL of each primer (0.4 μM), 8.5 μL of ultra-pure water, and 2 μL of template DNA. Positive controls included DNA from *Borrelia anserina* PL (GenBank code DQ849626), *Anaplasma platys* (OQ155255), and *Babesia* sp. “pudui” (ON994405). Ultra-pure water was used as a negative control. PCR products were stained with GelRed^®^ (Biotum, Tehran, Iran), subjected to horizontal electrophoresis in 2.0% agarose gels, and then visualized using an ENDURO™ GDS UV transilluminator (Labnet International, Edison, NJ, United States).

### 2.3 Ethical aspects

The procedures performed in this study were verified and approved by the Bioethics Committee of the School of Veterinary Sciences, Universidad de Concepción (CBE 47–2017; CBE-51-2019). The capture of rodents and field work in national parks and reserves were authorized by the Servicio Agrícola y Ganadero (SAG; #7034–2017, #7684/2017, #3731/2018, #8517/2018, #774/2019, and #1829/2020), and the Corporación Nacional Forestal (CONAF; Permits #045–2017 and #005–2019), respectively.

## 3 Results

A total of 207 rodents belonging to six species and four genera were captured (see Supplementary Table S2 for details). The most abundant species was *Abrothrix olivacea*, with 93 individuals, followed by *Abrotrix hirta*, with 77 individuals, and *Oligoryzomys longicaudatus*, with 31 individuals. The highest number of rodents were collected at Conguillio National Park and Sendero Bosque Piedra, with 47 rodents at each location (Supplementary Table S2). PCRs targeting the *GAPDH* gene produced amplicons of the expected size in all samples, confirming successful DNA extraction. All samples presented optimal DNA purity ratios (an A260/A280 DNA ratio within the 1.6–2.0 range). However, screenings for *Borrelia*, *Anaplasmataceae*, and Piroplasmida yielded negative results in all rodent samples while DNA in positive control was successfully detected.

## 4 Discussion

Recognizing infectious agents in wildlife is crucial for understanding disease dynamics, which is essential for both preserving biodiversity and preventing disease transmission to humans and domestic animals (34). Hence, continuous surveillance of these rodent species is imperative for public and animal health, as well as for ecosystem management (35).

To the best of our knowledge, this study represents the first assessment of the presence of DNA from *Borrelia* and piroplasmids, and the second assessment of *Anaplamataceae* bacteria in cricetid rodents from Central and Southern Chile, with screenings yielding negative outcomes among all cricetid rodent samples. The absence of DNA from these TBIAs in the surveyed rodents may arise due to multiple factors, including potential compromises in DNA extraction quality (32), the non-presence of DNA from screened TBIAs, or the lack of suitable vectors in the sampled areas (36, 37). However, the first statement was dismissed since all samples showed optimal DNA purity ratios, conducive to effective PCR amplification (32). This, coupled with successful internal control amplification, validated the DNA extraction process (33).

The non-detection of DNA of TBIA in the samples examined in this work contrasts with prior findings in other Chilean regions, where the DNA of *Borrelia*, members of the *Anaplasmataceae* family, and the Piroplasmida order have been reported in various tick genera, including *Ixodes* (16, 19, 38–40), and *Ornithodoros* (38, 41–43), as well as in their associated hosts (17–19, 39, 44, 45).

Regarding cricetid rodents, surveys in northern Chile have detected *Borrelia* DNA in blood samples from *Oligoryzomys longicaudatus* and *Phyllotis xanthopygus* collected in Bosque Fray Jorge National Park and Socoroma, respectively (17). Additionally, *Borrelia* DNA was detected in *Ixodes sigelos* group ticks on *Phyllotis darwini* and *Abrothrix longipillis* in Bosque Fray Jorge National Park and Isla Mocha National Park (38), and in *Ornithodoros* sp. ticks feeding on *P. darwini* in Río Los Cipreses National Reserve (41). In southern Chile, *Borrelia chilensis* DNA was found in *Ixodes stilesi* from *O. longicaudatus* in Valdivia (16). Collectively, these findings point to the prevalence of three genotypes and a unique genospecies, *B. chilensis*, within the Lyme *Borrelia* group, in addition to three genotypes from the Relapsing fever *Borrelia* group, thereby underscoring the *Borrelia* diversity associated with these rodents and their potential role as a reservoir host for the *Borrelia* species (16, 17).

Conversely, the detection of *Anaplasmataceae* DNA in cricetid rodents is limited, with records primarily involving “*Candidatus* Neoehrlichia chilensis” found in the tissue of *Abrothrix* rodents captured in the Corral commune in Valdivia (18), and in *I. sigelos* ticks from *P. darwini* in Bosque Fray Jorge National Park (38). Notably, DNA of “*Ca. N. chilensis*” has been predominantly detected in *Ixodes* ticks (46) and their associated hosts (18, 47). This suggests that “*Ca. N. chilensis*” could be transmitted by widespread *Ixodes* species among cricetid rodent populations in Chile. Thus, the involvement of *Abrothrix* rodents and *I. sigelos* in the epidemiology of “*Ca N. chilensis*” is highlighted (18, 38).

In 2022, the Piroplasmida order was associated with cricetid rodents in Chile for the first time (19); the DNA of *Babesia* was detected in the blood samples of *Abrothrix jelskii* collected in Parinatoca and *P. darwini* in Llanos de Challe National Park. Interestingly, despite being collected ~1,122 Km apart, two samples each from *A. jelskii* and *P. darwini* shared the same *Babesia* haplotype. This finding suggests a ubiquitous vector for this genotype, which is part of a novel *Babesia* group associated with South American small mammals (19, 48, 49). Additionally, a distinct *Babesia* genotype found in *P. darwini* from Llanos de Challe National Park aligns with the *Babesia microti* group, underscoring the emerging diversity of *Babesia* in Chilean cricetid rodents (45).

In addition to rodents, records of the evaluated TBIAs in wildlife in Chile include the detection of *A. platys* DNA in fox species *Lycalopex culpaeus* and *Lycalopex griseus* in central and northern Chile (44). Notably, *A. platys* primarily associates with ticks from the *Rhipicephalus sanguineus* group, which are common on canids (50). Other reports include DNA detections of *Babesia* sp. “Pudui” and the *Anaplasma phagocytophilum* “Patagonia” variant in both mainland and insular populations of the cervid *Pudu puda*, and in *I. stilesi* ticks that infest them in southern Chile (39, 45). Although *I. stilesi* commonly parasitizes the rodent *O. longicaudatus* (51) and is considered a potential vector of these infectious agents, the *Babesia* sp. “Pudui” and *A. phagocytophilum* “Patagonia” variant seem to have specific vertebrate host preferences (52, 53), primarily for *P. puda* in Chile (39, 45). This host specificity might account for our inability to detect the DNA of these TBIAs in *O. longicaudatus* samples.

The lack of TBIA DNA in our samples raises important hypotheses regarding the dynamics of these agents in the studied rodent populations: Is there an undetected low prevalence of the evaluated TBIAs in the sampled areas, or are these TBIAs absent in the surveyed rodent populations? Unpublished data from our sampling indicate a very low prevalence of ticks, a fact that supports the first hypothesis, given that the presence of the vector would imply the circulation of the TBIAs. Further studies focusing on the temporal and spatial variability of TBIAs in Chilean cricetid rodents, the frequency of these TBIAs in vectors, and the factors affecting the presence of these infectious agents in rodents will be insightful in answering these questions.

## Data availability statement

The original contributions presented in the study are included in the article/Supplementary material, further inquiries can be directed to the corresponding author.

## Ethics statement

The animal study was approved by the Bioethics Committee of the School of Veterinary Sciences, Universidad de Concepción: CBE 47–2017; CBE-51-2019. The study was conducted in accordance with the local legislation and institutional requirements.

## Author contributions

MA: Conceptualization, Writing – original draft, Writing – review & editing. RT: Investigation, Methodology, Writing – review & editing. AS: Conceptualization, Methodology, Writing – review & editing. MS-dF: Methodology, Writing – review & editing. SM-L: Funding acquisition, Methodology, Writing – review & editing. CL-A: Conceptualization, Funding acquisition, Methodology, Writing – review & editing. AH: Conceptualization, Methodology, Writing – review & editing.
